# Volume unbalance on the 2016 Amatrice - Norcia (Central Italy) seismic sequence and insights on normal fault earthquake mechanism

**DOI:** 10.1038/s41598-019-40958-z

**Published:** 2019-03-12

**Authors:** Christian Bignami, Emanuela Valerio, Eugenio Carminati, Carlo Doglioni, Pietro Tizzani, Riccardo Lanari

**Affiliations:** 1 Istituto Nazionale di Geofisica e Vulcanologia, Rome, Italy; 2 Dipartimento di Scienze della Terra, Sapienza University, Rome, Italy; 3 National Research Council (CNR), Istituto di Geologia Ambientale e Geoingegneria (IGAG), Rome, Italy; 4 National Research Council (CNR), Istituto per il Rilevamento Elettromagnetico dell’Ambiente (IREA), Napoli, Italy

## Abstract

We analyse the M_w_ 6.5, 2016 Amatrice-Norcia (Central Italy) seismic sequence by means of InSAR, GPS, seismological and geologic data. The >1000 km^2^ area affected by deformation is involving a volume of about 6000 km^3^ and the relocated seismicity is widely distributed in the hangingwall of the master fault system and the conjugate antithetic faults. Noteworthy, the coseismically subsided hangingwall volume is about 0.12 km^3^, whereas the uplifted adjacent volumes uplifted only 0.016 km^3^. Therefore, the subsided volume was about 7.5 times larger than the uplifted one. The coseismic motion requires equivalent volume at depth absorbing the hangingwall downward movement. This unbalance regularly occurs in normal fault-related earthquakes and can be inferred as a significant contribution to coseismic strain accomodated by a stress-drop driven collapse of precursory dilatancy. The vertical coseismic displacement is in fact larger than the horizontal component, consistent with the vertical orientation of the maximum lithostatic stress tensor.

## Introduction

Earthquakes produce dissipation of energy trapped by the pressure gradient forming between two walls moving at different velocity during the interseismic period. Regardless the tectonic setting, any motion along fault planes determines the shear between two volumes generating the double-couple mechanism^1^. Earthquakes modify the Earth’s surface, which can now be precisely measured by InSAR data. The original model of elastic rebound^2^ predicts symmetric displacement on both fault walls, consistent with observations from earthquakes occurring along strike-slip faults. The advent of GPS and Differential SAR Interferometry (DInSAR) data has proven to be essential to carry out a step forward in analysing earthquakes and their coseismic effects on the ground^3,4^. Along dipping faults, the recorded displacement is asymmetric because the hangingwall systematically moves more than the footwall. Ground deformation maps show that the displacement (either subsidence or uplift) of hangingwall blocks is larger than that of footwall blocks. In fact, normal fault-related earthquakes are characterized by larger coseismic hangingwall subsidence than correlated footwall uplift^5^; vice versa, along thrust faults, the hangingwall uplifts, whereas the footwall barely moves^6^. This is geologically obvious because in extensional geodynamic settings the dominating subsidence generates sedimentary basins, whereas the larger hangingwall uplift in contractional settings determines the growth of orogens, although extensional and compressional tectonics may occur also in areas characterized by regional uplift or subsidence^7^. Different types of energy accumulation have been proposed as a function of fault motion in favour or against gravity^8,9^. In fact, the number and duration of aftershocks is larger for extensional earthquakes with respect to the other tectonic settings, possibly because they work in favour of gravity^10^. In this paper, we compute the volumes characterized by uplift and subsidence during the extensional tectonics-related Central Italy 2016 seismic sequence and we speculate on a model explaining why the coseismically subsided volume is much bigger than the uplifted one. Is this phenomenology consistent with the coseismic horizontal elastic rebound or more suitable with the hangingwall gravitational collapse? We address the question whether the rock volume dilated in the brittle layer during the interseismic preparatory period was only elastically stretched^1^ or alternatively fractured and permeated by a population of thousands of microfractures^8^.

## Geologic and Geophysical Setting of the Amatrice-Norcia (Central Italy) 2016 Seismic Sequence

**Figure 1:**
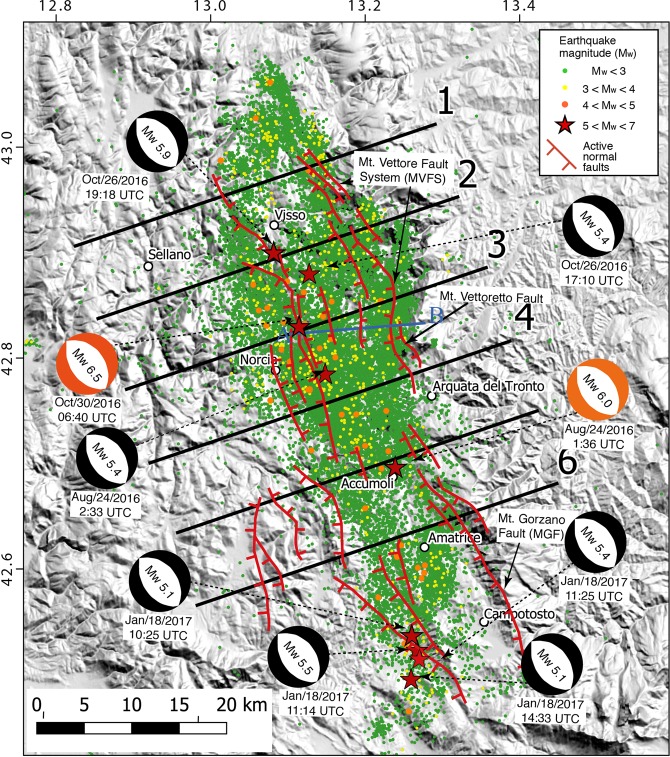
Map of the 2016 M_w_ 6.5 Amatrice-Norcia seismic sequence. 1 to 6 are the cross-sections of the seismicity and coseismic vertical motion shown in Fig. 2. (**A**,**B**) Is the trace of the cross-section shown in Fig. 3.

**Figure 2:**
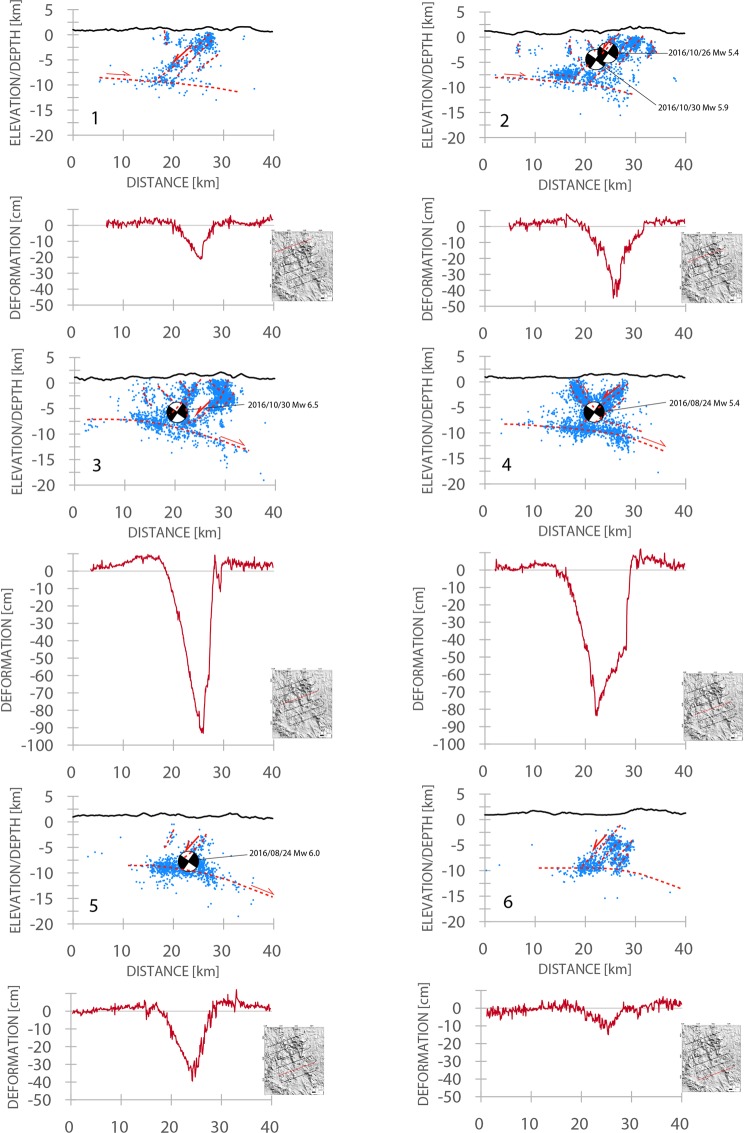
Cross-sections of the seismicity occurred during the 2016 Amatrice-Norcia seismic sequence with the associated vertical displacement recorded by SAR data. The dashed red lines represent the main inferred fault planes. The zero of the vertical displacement shown below each section represents the datum plane before the earthquake. Each section shows structural differences, illustrating the irregular shape of ruptures delimiting the prismatic volume of the graben or half-graben. In some sections, the SW-dipping master normal fault is associated with an antithetic NE-dipping conjugate faults. In all sections it occurs a low-angle NE-dipping decollement in which the overlying seismic volume is lying. The maximum coseismic subsidence developed in the central part of the sequence where the largest M_w_ 6.5 event occurred. Earthquakes data after^12^.

On August 24^th^ 2016, an M_w_ 6.0 earthquake (hypocentral depth at about 8 km) started a seismic sequence in Central Italy that had its apex with a M_w_ 6.5 (hypocentral depth at about 7 km) on October 30^th^. More than 100,000 aftershocks struck the area during the more than 30 months long and still active sequence^11,12^, including a M_w_ 5.9 event on October 26^th^, 2016 and other six 5.9 > M_w_ ≥ 5 events (Fig. 1; data from ISIDe working group, 2016; http://iside.rm.ingv.it/iside/standard/index.jsp and 12). In section, the seismicity illuminates a triangular volume with scattered clouds along a SW-dipping normal fault system dipping 45°–55° and conjugated NE-dipping faults. The faults tip between 6 and 10 km depth over a few km thick, 2°–15° NE-dipping low-angle normal faults system (Fig. 2).

The Apennines are in the hangingwall of a ‘westerly’-directed and ‘easterly’-retreating subduction zone. Contraction occurs in their eastern margin where the accretionary prism involves the shallow layers of the Adriatic plate. The axial part of the belt rather pertains to the backarc undergoing extension at 3–5 mm/yr rates due to the ‘eastward’ retreat of the slab hinge^13,14^. The accretion and the following extension affected the sedimentary succession of the Tethyan Mesozoic passive continental margin and the overlying active margin foredeep deposits^15–17^. The 2016 Amatrice-Norcia seismic sequence fits the extensional tectonics affecting the central Apennines fold-thrust belt since at least Pliocene time^18,19^ that generated a system of active NW-trending normal faults^20–22^. The seismic sequence activated progressively a crustal volume 70–90 km long, 10–15 km wide and about 8–10 km deep. The volume of the hangingwall prism affected by coseismic slip amounts to 5500–6700 km^3^. The wealth of good quality strong motion, seismological^11,12^, geodetic^3,23^ and geological data^24–29^ allows an unprecedented complete analysis of the boundary conditions in which the seismic sequence evolved.

The rupture model for the M_w_ 6.0 August 24^th^ event is characterized by three factors: (1) bilateral rupture^30^, (2) fast rupture velocity (3.1 km/s), and (3) pronounced heterogeneities of the slip distribution. At depth, the slip is characterized by two main patches, the southern one being smaller and characterized by maximum slip of ~100 cm, and the northern one with an average slip of ~60 cm^31,32^. The October 26^th^ M_w_ 5.9 earthquake is actually a double event and the two hypocenters are located at a distance of ~4 km. The rupture history of the October 30^th^ M_w_ 6.5 earthquake is characterized by rupture velocity of 2.7 km/s and by a large slip patch located ~5 km up-dip from the hypocenter with average slip of 130 cm and maximum slip of 260 cm^12^. The rupture reached the surface close to the mapped location of the Mt. Vettore–Mt. Bove fault system with further displacement along the ‘nastrino’ of ~50 cm (in places up to ~200 cm)^33^. No coseismic slip was observed along the Mt. Gorzano fault during the entire seismic sequence.

Paleoseismological studies focused on the Mt. Vettore Fault System and on the Mt. Gorzano Fault showed that medium-high magnitude earthquakes (M_w_ ≥ 6.0) struck these zones over the past centuries^34^. In particular, since the 17^th^ century, several moderate-to-large earthquakes affected the Amatrice-Accumoli area, one occurred in 1627 near the town of Accumoli (M_w_ ~5.3) and one that struck the town of Amatrice in 1639 (M_w_ ~6.2), provoking numerous casualties and damage^35^.

The Norcia area was also affected by the 1730 (M_w_ ~6) and 1979 (M_w_ 5.8) earthquakes, which generated surface faulting^34^. This area was also affected and shaken by several earthquakes that occurred in nearby regions, such as the 1703 Valnerina-L’Aquila seismic sequence (M_w_ ~6.8, 33), the 1997 Colfiorito earthquake (M_w_ 6.0)^36^ and the 2009 L’Aquila earthquake (M_w_ 6.3)^37,38^. The 2016 seismic sequence filled the seismic gap separating the 1997–1998 Colfiorito sequence (M_w_ 5.4 and M_w_ 6.0 earthquakes) and the M_w_ 6.3 2009 L’Aquila earthquake. The hypocentre of the first mainshock (M_w_ 6.0 August 24^th^, 2016) was located about 10 km north of Amatrice^12^. One hour later an M_w_ 5.4 earthquake in the Norcia area followed this event. The earthquake occurred along a 50° SW-dipping and ~ 20–25 km long extensional fault^31,39^. On October 26^th^, a M_w_ 5.9 earthquake nucleated 25 km to the north, near the village of Visso, activating another segment of the normal fault. Four days later, on October 30^th^, the largest mainshock of the sequence (M_w_ 6.5) hit the area in between the two previous events. The hypocentre was located at about 7 km along a 55° SW dipping fault^12^.

The distribution of aftershocks (Fig. 2) suggests the activation, during this seismic sequence, of several secondary antithetic NE-dipping extensional faults below the Norcia basin at shallow depth (<4 km). The M_w_ 5.4 event following the August 24^th^ 2016 M_w_ 6.0 event, nucleated at the intersection between an antithetic structure and the main fault and was probably located on the first one^11^. The whole normal fault system, confined within the first 10 km of the upper crust, is detached and floored by a shallow, gently 10°–15° easterly-dipping and 2–3 km-thick layer in which small events plus a series of large extensional aftershocks (≈M_w_ 4.0) occurred, possibly located in the Triassic evaporitic layers^26,40^.

According to the seismological data, the entire sequence activated a SW-dipping normal fault system, striking about N150°–160° and dipping ~45°–55°, locally with listric shape. Possible reactivation and inversion of an inherited W-dipping thrust has been advocated^41^. These principal faults were recognized as the Mt. Gorzano Fault and the Mt. Vettore fault systems^31,39,42^, both characterized by extensional/transtensional kinematics and dissecting the heterogeneous clayey/marly to carbonatic sedimentary succession of the central Apennines^40^. The Mt. Gorzano extensional fault is ~ 30 km long, dips ~ 60° to the SW and accommodates a maximum down-dip displacement of ~2.3 km^42^. Where exposed, the fault juxtaposes Early-Middle Miocene marly limestones (Marne con Cerrogna Fm) in the footwall with Messinian siliciclastic deposits (Laga Fm) in the hangingwall.

**Figure 3:**
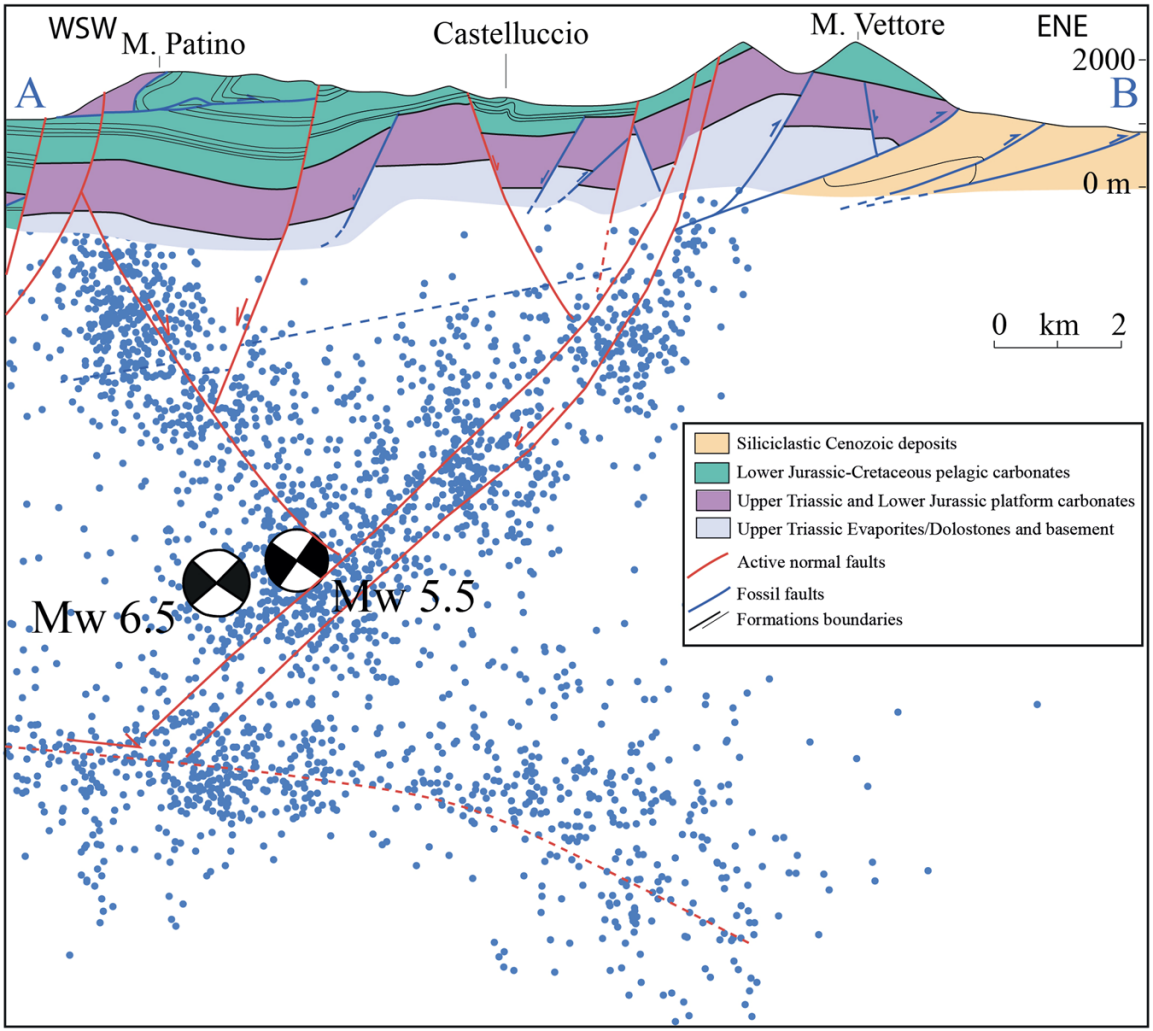
Geological cross-section of the area affected by the M_w_ 6.5 Amatrice-Norcia earthquake. Geological data after^67^.

**Figure 4:**
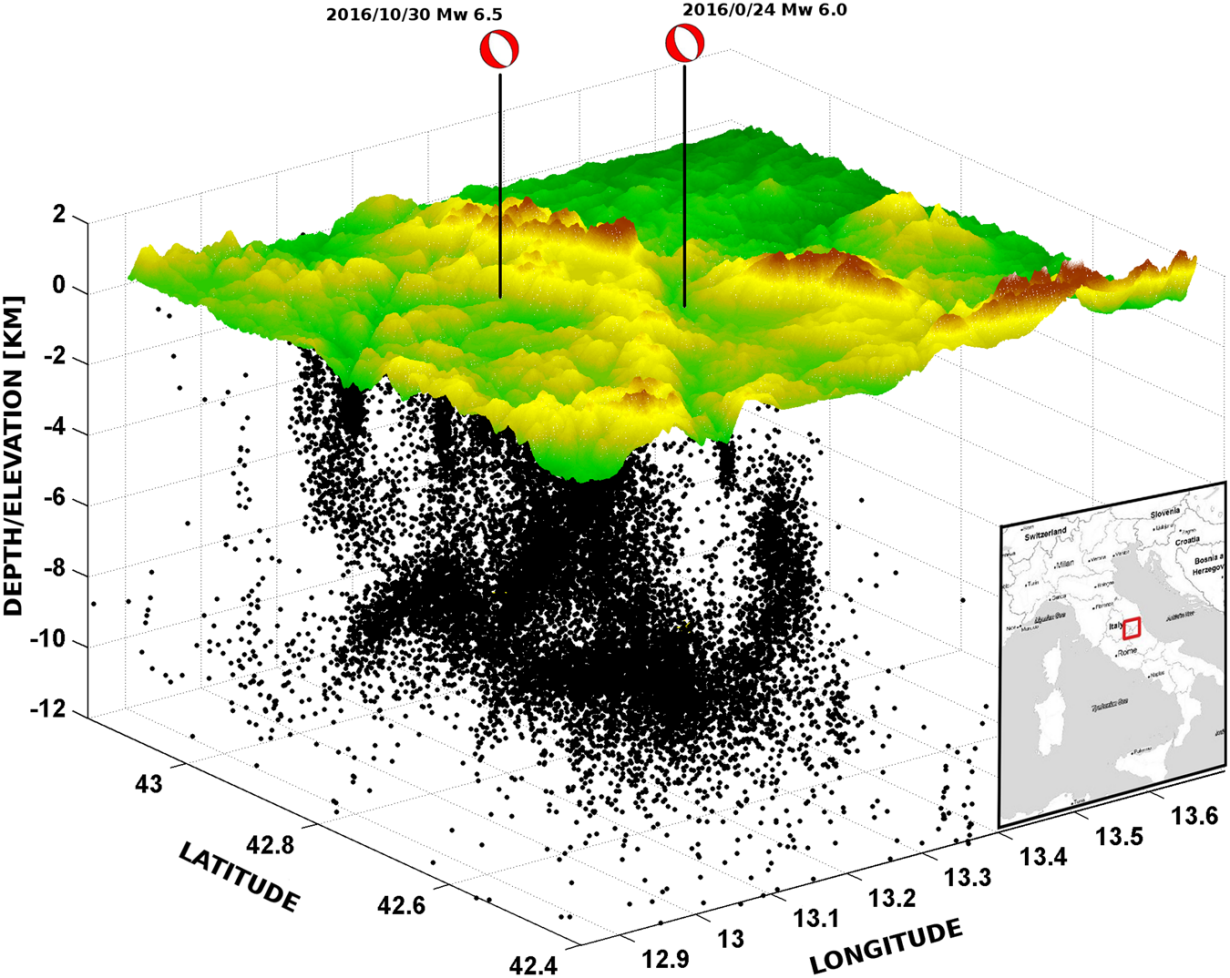
3D view of the seismicity related to the 2016 Amatrice-Norcia seismic sequence. The figure shows the spatial distribution of the more than 100,000 aftershocks and the focal mechanisms of the two M_w_ 6.0 and M_w_ 6.5 mainshocks occurred on August 24^th^ 2016 and October 30^th^ 2016. The mechanisms show N150°–160° trending normal faulting. The area affected by the sequence is elongated NW-SE, about 70–90 km long and 10–15 km wide. The distribution of the seismicity demonstrates the shape of the involved upper crustal volume rather than a simple planar fault.

The coaxial Mt. Vettore fault system is ~18 km long and consists of a series of SW-dipping (34°–75°) extensional faults, cutting through the flanks and the foothills of Mt. Vettore, Mt. Porche, and Mt. Bove; a maximum total vertical displacement of ~1.2 m was reconstructed along the Mt. Vettore Fault segment^34,43^. The antithetic conjugate NE-dipping normal fault is not always illuminated by seismicity, and coseismic deformation of the graben or half graben continuously varies moving along strike (Fig. 2), describing a prismatic volume with clouds of earthquakes along the main fault planes (Figs 3 and 4).

## Space Geodesy Results

**Figure 5:**
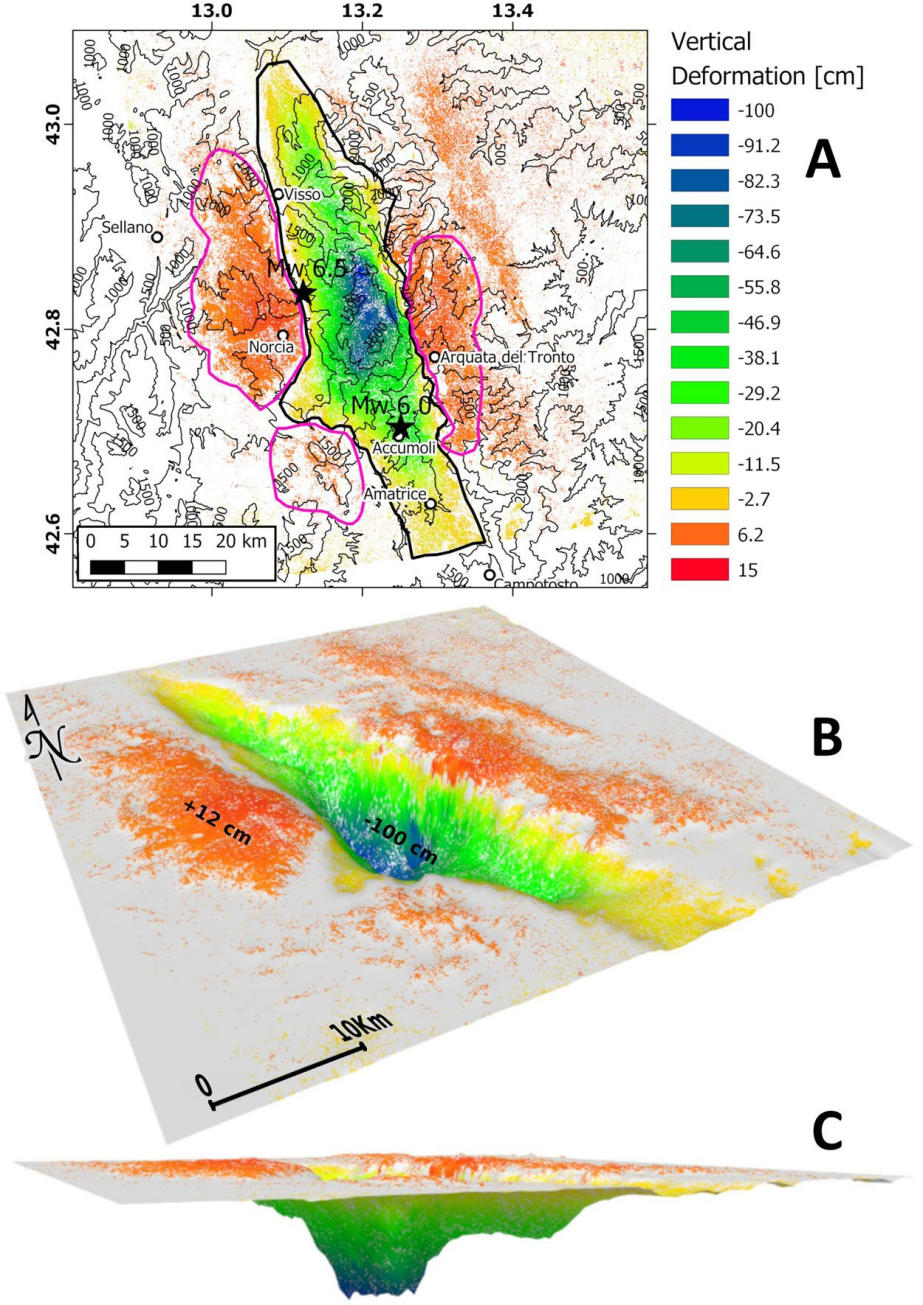
(**A**) Map showing the cumulated displacements occurred from September 2015 and November 9, 2016. It is recorded by the ALOS2 DInSAR data, showing the areas collapsed and uplifted during the seismic sequence between M_w_ 6.0 August 24^th^ and M_w_ 6.5 October 30^th^ 2016 assuming that no pre-seismic deformation occurred. Coseismic uplift is marginal with respect to subsidence. The largest deformation is concentrated in the hangingwall of the master WSW-dipping normal fault system. Maximum coseismic subsidence was around 100 cm, whereas the highest uplift in the hangingwall (i.e., to the west) was about 10–12 cm. The estimated collapsed volume is 0.12 km^3^. The uplifted volume in the hangingwall is about 7.5 times smaller, posing the question of the unbalance of the volumes. According to error estimates (see method section), the values ranging between −3 and 3 cm are masked out. The dashed black and magenta polygons refer to the areas selected for subsided and uplifted volume calculation, respectively, reported in Supplementary Information Tables S1 and S2. (**B**,**C**) are 3D views of the vertical deformation map (**A**). The vertical exaggeration is 5000 times. The grey areas refer to the masked-out deformation values ranging between −3 cm and 3 cm (see main text), and colour code is equal to 2D map in (**A**). The subsided volume has a depocenter in the center of the asymmetric graben. The subsided volume is much larger than the uplifted one.

**Figure 6:**
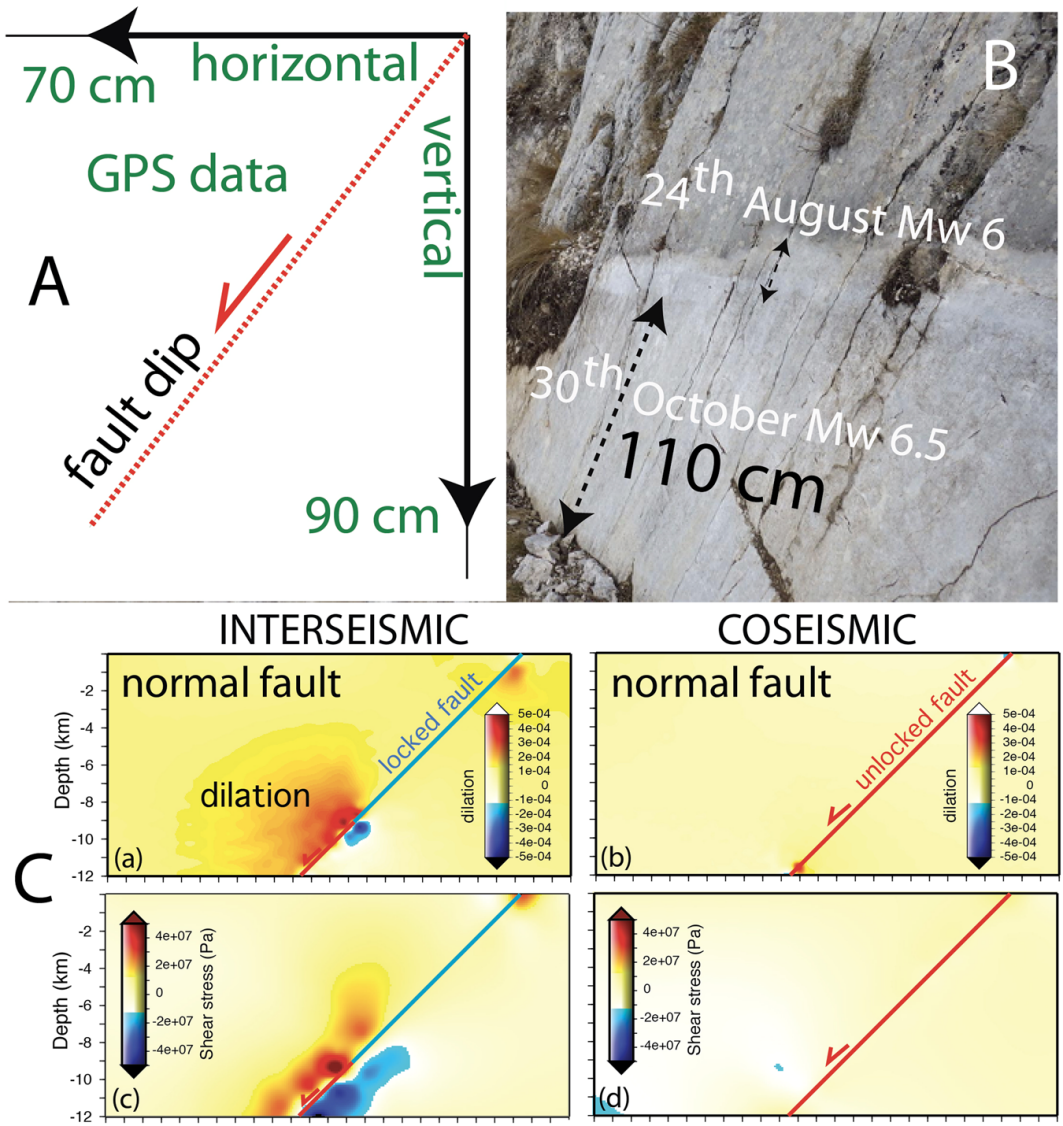
(**A**) Horizontal and vertical components associated with the October 30^th^ 2016 M_w_ 6.5 mainshock (data after^4^). Notice the larger coseismic vertical displacement, in agreement with the vertical maximum stress tensor (σ_1_). The fault dip correctly represents the vector sum of the horizontal and vertical components of the displacement. (**B**) Surface rupture associated with the two seismic events^68^. (**C**) Numerical modelling of interseismic and coseismic deformation in a simplified brittle upper crust and visco-plastic lower crust. During the interseismic the fault is locked in the upper crust, whereas is shearing in steady state in the lower crust. A dilated volume forms above the brittle-ductile transition to accommodate the strain partitioning. At the coseismic stage, the fault hangingwall collapses and recover the previously formed dilation. See shear stress associated with the two stages (modified after^50^).

Figure 5 shows the cumulative vertical displacement from August 24^th^ until November 2016 in the area affected by the seismic sequence. These motions sum up the effects of the Amatrice-Norcia 2016 part of the seismic sequence. The coseismic uplift of the hangingwall is marginal, both in amplitude and spatial extent, with respect to the dominant subsidence. The DInSAR-related vertical deformation map (Fig. 5) shows a subsidence peak of almost 100 cm in the central part (blue), and the average amount of subsidence is about 24 cm. On the contrary, uplift does not exceed 14 cm. Note that we masked out the areas characterized by values ranging between −3 and 3 cm, with 3 cm corresponding to about 1/8 of the exploited ALOS2 SAR system wavelength and representing a realistic error range for the estimated co-seismic displacement. Moreover, the horizontal coseismic motion equals to the cosine of the fault dip, whereas the vertical motion represents the relative sine. The coseismic horizontal motion is smaller than the vertical component, confirming that subsidence is constrained by the vertical maximum stress, i.e., the lithostatic load. The fault dip is the vector sum of the vertical and horizontal components (Fig. 6).

**Figure 7:**
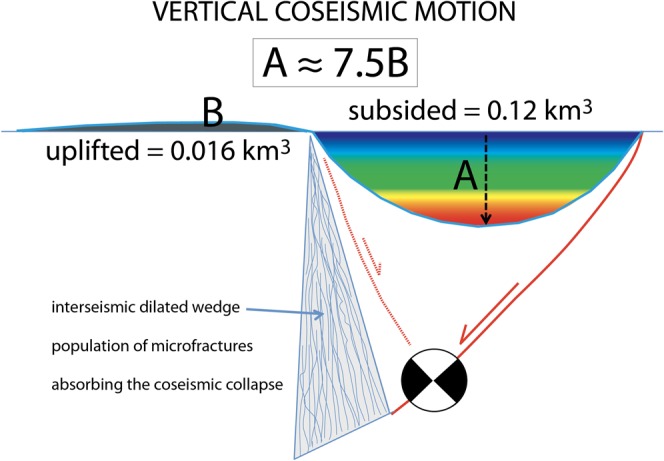
During the 2016–2017 Amatrice-Norcia sequence, the subsided volume (**A**) was about 7.5 times larger than the uplifted volume (**B**). This volume unbalance can be explained either by an elastically stretched crust or alternatively permeated by a large number of fractures formed during the interseismic period in the brittle upper crust. This would require the existence at depth of a dilated volume able to accommodate the fall of the normal fault hangingwall. The collapse of the overlying prismatic volume may be triggered by the final loss of strength in the dilated wedge and along the fault.

DInSAR vertical displacement data allowed also the computation of the rock volumes displaced during the whole seismic sequence. We used two different methods (see method section) for such computation to crosscheck the validity of results. As expected, we found that subsided and uplifted volumes are largely different: the volumes amount to 0.12 km^3^ and 0.016 km^3^, for subsidence and uplift, respectively. This unbalance is clearly highlighted in Fig. 5, where a 3D view of the deformed surface is showed. The difference between subsided and uplifted volumes implies the presence at depth of a crustal zone able to accommodate the hangingwall settlement (Fig. 7). In particular, this missing volume occurs at the coseismic phase, hence it can be considered instantaneous, and responsible for permanent, i.e., inelastic, deformation within the involved rocks. We infer that this inelastic deformation should take place at depth allowing the rock volume to recover a previously dilated zone. Two models have been advocated to explain the occurrence of this pre-existing dilated zone and the coseismic phenomenology, i.e., the horizontal interseismic elastic stretching and the fault motion (associated with elastic rebound) generating the earthquake, or the interseismic stretching associated with the generation of a population of microfractures in the upper crust, eventually determining the gravitational collapse of the hangingwall and normal fault motion with the related double couple.

## Elastic Rebound Versus Gravitational Collapse

It is commonly stated that earthquakes represent the sudden elastic rebound dissipating the pressure gradient accumulated during the interseismic period. This model fits most of the data related to strike-slip and contractional earthquakes. However, the phenomenology associated with normal fault earthquakes shows a number of misfits with respect to the elastic rebound model. Although faults are not straight planes, but rather undulated surfaces and their motion may affect a number of sub-parallel faults bounding a volume rather a single planar fault, the simplified Okada model^44^ correlates quite correctly the surface motion when fault dip and slip generated at depth by an earthquake are imposed. However, the Okada model does not analyse the mechanism generating earthquakes, but only reproduces the displacement associated with slip on a discrete fault plane within a half-space in an infinite medium. We discuss here whether, in an extensional tectonic setting, the surface deformation described by the Okada^45^ model and fitting coseismic observations is more consistent with a model in which the energy accumulated during the interseismic period is elastic or gravitational. At the coseismic stage, both models generate fault motion, double-couple mechanism, elastic waves generation, deformation of the surrounding volume, but they differ in a number of further constraints, such as interseismic fracturing e fluids motion, coseismic shear heating, volume folding, hangingwall motion, etc.

As summarized in Fig. 7, during the Amatrice-Norcia sequence the collapsed volume (A) has been about 7.5 times larger than the uplifted volume (B). This unbalance has been explained as a temporary setting, gradually compensated during the post-seismic deformation by the footwall uplift of the viscous-plastic deformation in the lower crust^5^. However, in the Apennines, there are several examples of coseismic surface rupture that formed at the mainshock and since then are crystallized with no further significant movement between footwall and hangingwall^25^, although other studies with newer techniques suggest that some post-seismic relaxation and footwall uplift may occur^5^.

In spite of the crustal-lithospheric extensional setting, at depth deeper than about 1 km, dilation occurs under a compressional horizontal stress due to lateral confinement of rocks. Therefore, horizontal σ_3_ is contractional at depth even in extensional tectonic environments. Horizontal stretching decreases progressively σ_3_ during the interseismic stage, whereas σ_1_ remains almost constant; thus, differential stress increases in time during the interseismic period, eventually leading to rupture, fracturing rocks under dilatancy. The question is whether this pre-earthquake dilated volume during the interseismic period was elastically stretched or it was permeated by a population of thousands of mm-scale microfractures as those that are routinely visible in fractured outcrops or in industrial boreholes. However, once rocks are stressed above their yield stress during the interseismic stage, fractures occur, decreasing or eliminating rocks elasticity.

**Figure 8:**
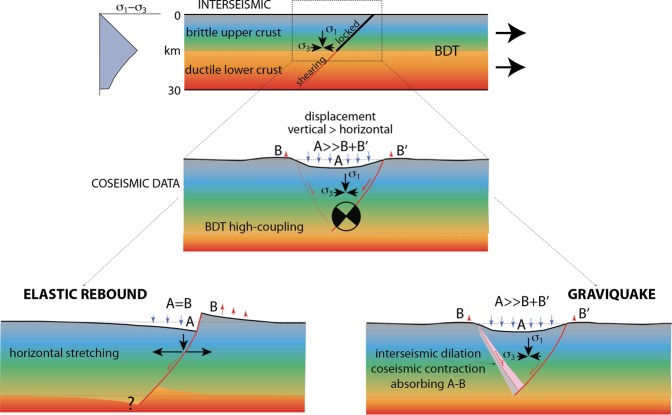
Comparison between two models to explain the phenomenology associated with normal fault-related earthquakes. Normal fault activates either by elastic rebound due to the crustal stretching (lower left) or to the gravitational collapse of the hangingwall (lower right). See text for further explanation. Notice that the elastic rebound is at odds with a number of observed data, such as (i) the larger vertical coseismic displacement with respect to the horizontal component, (ii) in spite of the extensional setting, the crust is in compressional state of stress, while the elastic rebound model requires horizontal interseismic stretching; (iii) at the bottom of the normal fault there is both positive and negative volume unbalance where the ductile crust cannot absorb instantaneously such deformation, particularly where the largest differential stress is required at the brittle-ductile transition (BDT); (iv) the coseismic and post-seismic symmetric footwall uplift and hangingwall subsidence (A = B) do not occur. These inconsistencies could rather be satisfied by the existence of a pre-existing dilated wedge formed during the interseismic period, which will eventually loose strength, allowing the hangingwall to collapse gravitationally and explaining the larger collapsed volume with respect to the uplifted one. Near fault black arrows indicate the state of stress required by the two compared models.

In nature, along dip-slip faults deformation is highly asymmetric, being mostly confined in the hangingwall. This implies a specular volume asymmetry at depth in order to accommodate the surface variation. In other words, the section should be balanced in terms of involved volumes. However, at the bottom of the brittle upper crust, there occurs the largest differential stress required to deform rocks at the brittle-ductile transition (BDT) and the ductile crust cannot absorb instantaneously such deformation (Fig. 8). Moreover, due to the higher strength, the BDT cannot represent a decoupling layer. This favours the hypothesis of the occurrence of microfractures in the ‘missing’ volume in the brittle crust itself. In addition, the larger movement recorded by GPS and DInSAR analyses is along the vertical^3–5^, following the lithostatic load that tends to match the maximum stress (σ_1_) in extensional tectonic setting, although the horizontal components (σ_2_ and σ_3_) are not negligible. Eventually, once rocks are stressed above their yield stress during the interseismic stage, fractures occur, decreasing rocks elasticity. The horizontal coseismic motion does better fit the gravitational model because the main hangingwall displacement is downward and not horizontal, as testified by SAR and GPS data. Coherently as well known in the literature, normal fault earthquakes increase their magnitude with the dip of the normal fault^8^.

We propose a scenario in which the upper crust is permeated by millimetric fractures, similar to those that are commonly detected by hydrocarbon wells^46^. The microfractures that form during the interseismic period might be partly filled by cement and partly by fluids as shown in hydrocarbon exploration boreholes, as also predicted by analogue models^47,48^ and suggested by multi-temporal SAR measurements^49^ as well. The interseismic dilatancy is documented both by well logs and was reproduced with numerical modeling^50^. Coseismic dilatancy and fracturing is also documented, particularly at fault tips of strike slip faults^51^. Dilatancy varies as a function of the tectonic setting and the interseismic, coseismic and postseismic period^50,51^.

Different fluids behaviour has been documented as a function of the extensional or contractional tectonic setting^52,53^. The fluids expulsion during the late pre-seismic and coseismic stage of the Amatrice-Norcia sequence^54^ supports the presence (at late interseismic stages) and the disappearance (during the earthquake) of a diffuse permeability, compatible with the occurrence and the partial closure during the coseismic stage of a multitude of microfractures. A similar behaviour of fluids expulsion was observed during the 2009 L’Aquila earthquake and is typical of normal fault-related earthquakes^53,55,56^. Fluids react at the coseismic stage^57^, being squeezed out of fractures while the hangingwall collapses, i.e., shrinking a previously dilated volume at depth^53^.

Figure 8 shows a model characterized by the interseismic generation of a dilated wedge permeated by microfractures in the brittle layer, above the ductile steady-state shearing layer. The BDT controls the strain partitioning and the switch between portions of the fault characterized by stick-slip behaviour in the upper brittle crust and continuous creep in the viscous-plastic lower crust. When the strength of the wedge and of the fault will not be any more able to sustain the weight of the wedge overlying the dilated volume, the hangingwall will collapse, generating the double couple related to the shear along the master fault plane, releasing the elastic waves of the earthquake. The slip along the fault allows the downward motion of the hangingwall that will close open fractures in the dilated wedge, generating the expulsion of fluids permeating the hangingwall. Based on rock-mechanics arguments, we interpret this pre-earthquake dilation as associated with microfractures that close catastrophically during the coseismic stage. Support to the occurrence of fractures is further provided by the discrepancy between the larger coseismic slip at the hypocenter (around 260 cm) and the coseismic surface fault displacement (90 cm). This difference implies a vertical coseismic stretching of the hangingwall, which can gradually recover the sudden dilation during the post-seismic gradual gravitational adjustment, being responsible for the more than 100,000 aftershocks up to early December 2018.

According to the model and to numerical simulations^8,50,58^, the fault hangingwall can collapse, favoured by gravitational energy stored during the interseismic period (Fig. 8), slipping along the fault plane. When the stresses related to gravitational energy exceed the strength of the faults and of the dilated zone, the crustal prism slips down along the normal fault system.

## Discussion and Conclusions

The August 24^th^ 2016, M_w_ 6.0 extensional earthquake started the Amatrice-Norcia (Central Italy) seismic sequence, reaching its mainshock the M_w_ 6.5 October 30^th^ at about 8–10 km depth. Based on geologic, seismologic and DInSAR data, a crustal volume of about 6000 km^3^, confined by a 45°–55° WSW-dipping fault system and its conjugate antithetic faults, subsided about 100 cm in the depocentre of the asymmetric graben. GPS data of the mainshocks constrain smaller horizontal displacements relative to the vertical coseismic motion, consistent with the normal fault dip.

We compare the elastic and gravitational models to explain the asymmetry and the volume unbalance. However, these aforementioned observations are consistent with a preparatory phase during the interseismic period in which a dilated volume accommodates the strain partitioning between the brittle and the viscous-plastic portions of the crust, characterized by stick-slip and steady state creep, respectively^50^. The entire sequence appears more coherent with the gravitational adjustment of the hangingwall of the fault system. The double-couple visible in the moment tensor is required and implicit with the downward motion of the hangingwall along the main normal fault surface. As interpreted in sections 1–6 of Fig. 2, the seismicity seems to depict the ‘extensional allochthon’^59^ where a combination of listric and rotating “book-shelf” faults accommodate horizontal extension above a low-angle detachment. However, the family of WSW-directed normal faults across the Apennines does not show any appreciable rotational evolution. In the example discussed in the article, the decoupling does not occur at the BDT, but within a shallower evaporitic layer. This layer acts as a boundary limiting both interseismic and coseismic evolution. This stratigraphic setting prevents the activation of larger upper crustal volumes, hence lowering the expected maximum earthquake magnitude. In fact, in the graviquake model^8^ it was pointed out that the ratio between the seismic volume depth and the activated volume and related fault length is about 1/3. Therefore, the occurrence of a bounding decoupling layer at about 8–10 km depth limits the volumes length that can be mobilized at each time at no more than 24–30 km, inhibiting a single fault 80–90 km long rupture, hence decreasing the maximum potential earthquake magnitude.

What can be observed during the 2016 Central Italy seismic sequence is that rupture propagated along strike of the volume axis and of the normal faults; different volumes collapsed activating different segments of the normal faults system, moving back and forth from south to north, and then south again. This evolution is compatible with the gravitational coseismic subsidence, although the pre-requisite is the occurrence at depth of an interseismically dilated zone able to be contracted and absorbing the volume downward motion.

Therefore, the analysis of the 2016 Amatrice-Norcia seismic sequence supports the following statements:

- Space geodesy (DInSAR) data show that the volume characterized by coseismic hangingwall subsidence is at least 7.5 times bigger than that coseismic uplift both in the hangingwall and in the footwall.
- The volume unbalance can be interpreted as the evidence in the brittle upper crust of a dilated wedge with a population of microfractures generated during the interseismic period.
- The gravitational collapse of the normal fault hangingwall could be constrained by the loss of strength of the dilated wedge and the normal fault, closing pre-existing microfractures, as postulated by the graviquake model^8^.
- In order to accommodate the coseismic displacement, the elastic rebound model would rather require a larger horizontal coseismic component and at the lower tip of the normal fault a volume unbalance that cannot be absorbed instantaneously due the higher strength at the BDT.

The larger collapsed volume confirms the asymmetric displacement and larger hangingwall motion^60^. We infer how this observation can be explained by an equivalent dilation generated at depth during the interseismic period, able to accommodate the fall of the hangingwall at the coseismic stage. All these data could rather be satisfied by the existence of a pre-existing dilated wedge in the upper brittle crust formed during the interseismic period, which will eventually loose strength allowing the hangingwall to collapse gravitationally. Moreover, in spite of the extensional setting, the crust is in a contractional state of stress, while the elastic rebound model requires horizontal stretching. This phenomenology suggests that earthquakes dissipate energy interseismically accumulated not only along the fault but rather stored within the volume that is eventually mobilized and deformed at the coseismic stage; the fault plane is the passive mechanical discontinuity where part of the gravitational energy is transformed into channelized elastic waves.

There are fundamental differences from a landslide as those analysed by^61,62^ and the proposed graviquakes model. First of all, the basal detachment plane of a landslide is emerging at the Earth’s surface (regardless it is submarine or subaerial), whereas in normal faulting is not, being the lower segment of the normal fault tipping and confined in the lower upper crust. Secondly, in a landslide the hangingwall is unconstrained and free to move relative to the atmosphere, allowing the volume to partial or full disaggregation, whereas a gravity driven normal fault hangingwall is fully confined by the neighbouring crustal volume. Therefore, even if landslides and normal faults are fuelled by gravity, they show very different geometry, kinematics and general phenomenology; consequently, the seismic record must be different (e.g., two-lobe vs. four-lobe seismic radiation pattern from a double couple source).

This research shows how elastic and anelastic deformation is partitioned during the seismic cycle of normal faults and could provide a framework for further investigations. The search for dilated wedges in the upper crust through magnetotelluric techniques, V_p_/V_s_ etc. could illuminate fluid-rich, low-resistivity volumes, which may help to recognize more seismically-prone active volumes.

## Methods

### DInSAR data

Using data obtained by the interferometric processing of the acquired SAR images (Supplementary Information Table S1), we computed the rock volumes coseismically affected by uplift and subsidence. All the SAR images were processed by means of classical DInSAR technique^63^, and we took the advantage of the SRTM 1-arc second DEM to remove phase topography. As typically performed from the combination of ascending and descending SAR dataset^6^, we can obtain the vertical and the east-west displacements of the ground deformations caused by the entire sequence.

In detail, we used two approaches in order to crosscheck the validity of the obtained volume estimates. The dataset consists of a pair of ALOS-2 SAR images acquired on ascending orbits on September 9^th^, 2015 and November 2^nd^, 2016, together with a pair of images on descending path, dated May 25^th^, 2016 and November 9^th^, 2016. The two ground deformation maps were then combined to obtain the vertical displacement map (Fig. 5).

The estimation of uplifted and subsided volumes requires the discrimination of positive and negative values of vertical deformation. Such discrimination was performed by defining a threshold to separate positive and negative values of deformation. The threshold was calculated by setting a range of values through the estimation of the error of the deformation map. The latter can be evaluated looking at the interferometric coherence of the used image pairs^64^, and such error was estimated (conservatively) around 3 cm. Therefore, after unwrapping interferometric data, we masked out the pixels ranging between −3 and 3 cm, setting to NaN deformation the data within these thresholds. All the negative values appear well separated in the central part of the map in Fig. 5. Such data were used for the rock volume of subsidence. As far as the uplifted volume, the selection of the footwall part is a little bit more difficult. Indeed, as showed in Fig. 5, there are several pixels that overcome the 3 cm threshold, and the identification of those pixels belonging only to the footwall uplift is not very easy. In order to overcome this point, we exploited the information reported in Supplementary Information Table S1, and a polygon that roughly separate pixels in the footwall was drawn (see Fig. 5).

Once the areas for subsided and uplifted volumes are selected, we can calculate the rock volume mobilized during the seismic sequence. It is worth noticing that data were also interpolated to fill-in the no data areas due to interferometric coherence loss.

The first adopted approach is an automatic one, implemented in Surfer® software. It uses a numerical integration algorithms proposed in^65^, the Extended Simpson’s 3/8 Rule, represented by the following formula:

1 $${\rm{Volume}}\approx \frac{3{\rm{\Delta }}y}{8}[{{\rm{A}}}_{1}+3{{\rm{A}}}_{2}+3{{\rm{A}}}_{3}+2{{\rm{A}}}_{4}\ldots +3{{\rm{A}}}_{{\rm{nCol}}-1}+{{\rm{A}}}_{{\rm{nCol}}}]$$

where Δy is the grid row spacing and ${\rm{A}}$ is the area derived from the following equation:

2 $${{\rm{A}}}_{{\rm{i}}}\approx \frac{3{\rm{\Delta }}x}{8}[{{\rm{G}}}_{{\rm{i}},1}+3{{\rm{G}}}_{{\rm{i}},2}+3{{\rm{G}}}_{{\rm{i}},3}+2{{\rm{G}}}_{{\rm{i}},4}\ldots +3{{\rm{G}}}_{{\rm{i}},{\rm{nCol}}-1}+{{\rm{G}}}_{{\rm{i}},{\rm{nCol}}}]$$

where ∆x is the grid column spacing and G_i,j_ is the grid node value in row i and column j.

A second, manual and simpler method was also adopted to estimate the involved volume. Such a method is based on the extraction of pixels from the vertical deformation map, outside the −3 cm ÷ 3 cm range, and summing the values of volumes for each pixel taking into account the area of the pixel itself, i.e., 900 m^2^ that corresponds to pixel size of the interferometric product. By easily summing all the selected pixels in a GIS environment (again considering the uplifted and subsided areas in the polygons), we can estimate the rock volumes involved during the seismic sequence. The results of the volume estimates are reported in Supplementary Information Table S2.

The collapsed volumes we computed with the two approaches are identical, therefore, the final estimate is set to 0.12 km^3^. The uplifted volumes are equal, too, and the final estimation is set to 0.016 km^3^, hence the final ratio between subsidence and uplift volumes mobilization is 7.5. Therefore, regardless the mechanism (elastic or inelastic) this observation requires at the coseismic stage the closure, at depth, of a dilated volume, equal to the difference between collapsed and uplifted volumes, able to absorb the fall of the hangingwall.

### GPS data

GPS data have a greater accuracy on the horizontal motion with respect to the vertical motion and for this reason they perfectly integrate with DInSAR data that have better and widely distributed vertical accuracy. The dense GPS network of the area allowed to record coseismic deformation quite in detail^3^: near Accumoli (Fig. 5), the 24^th^ August subsidence and SW-motion has been 17 cm and 5 cm respectively, whereas the 30^th^ October, the recorded subsidence and horizontal motions have been about 40 cm and 25 cm toward the SW, respectively^3^. Footwall uplift has been of 2–5 cm.

During the October 30^th^ M_w_ 6.5 Norcia mainshock, at the Mt. Vettore Fault, ~90 cm of hangingwall coseismic vertical component and ~70 cm of westward horizontal component were recorded^4^. These movements demonstrate that the vertical component is larger than the westerly directed horizontal motion, being their value constrained by the ~50° fault dip plane.

In order to figure out any possible early post seismic deformation, we collected GPS time series from the measurement network in our study area^66^. Indeed, it is well known that DInSAR displacement maps are static pictures of all the deformation occurred between the master and slave images used to perform the interferometric processing. In particular, the map depicted in Fig. 5 accounts for all the displacements occurred from September 2015 and November 9, 2016. Therefore (assuming that no pre-seismic signals are present), any post-seismic deformation has occurred after the main events (i.e., August 24^th^, October 26^th^, and October 30^th^, 2016) should be embedded in the DInSAR map. Unfortunately, most of the GPS stations in the affected area, and overlapping the SAR map, have been installed after the seismic sequence, hence we are not able to discriminate between co- and post-event displacements because GPS time series start since November 10^th^–14^th^ 2016 (Supplementary Information Fig. S1).

## Supplementary information

Supplementary Information
