# Supplementary material for: Volume unbalance on the 2016 Amatrice - Norcia (Central Italy) seismic sequence and insights on normal fault earthquake mechanism
Source: Sci Rep. 2019 Mar 12;9:4250. doi: 10.1038/s41598-019-40958-z (PMC6414550; doi:10.1038/s41598-019-40958-z)
Supplement: Supplementary file 1 — Supplementary Information [file 41598_2019_40958_MOESM1_ESM.pdf]

Supplementary information for "Volume unbalance on the 2016 Amatrice - Norcia (central Italy) seismic sequence and insights on normal fault earthquake mechanism"

Christian Bignami[a], Emanuela Valerio[b], Eugenio Carminati[b,c], Carlo Doglioni[a,b*], Pietro Tizzani[d], and Riccardo Lanari[d]

[a]Istituto Nazionale di Geofisica e Vulcanologia, Rome
[b]Dipartimento di Scienze della Terra, Sapienza University, Rome, Italy
[c]National Research Council (CNR), Istituto di Geologia Ambientale e Geoingegneria (IGAG), Roma, Italy
[d]National Research Council (CNR), Istituto per il Rilevamento Elettromagnetico dell'Ambiente (IREA), Napoli, Italy

Corresponding Author:
Carlo Doglioni, Via di Vigna Murata 605, 00143 Rome, Italy
email: carlo.doglioni@uniroma1.it

Satellite	Interferometric pair	Orbit	Sensor Look Angle in the epicentral area (deg.)
ALOS-2	2015/09/09-2016/11/02	ASC	36.3
ALOS-2	2016/05/25-2016/11/09	DESC	40.5

Table S1. Available SAR images for interferometric processing.

Approach	Volume Type	Volume
Automatic approach	Subsidence	0.11889 km^3
	Uplift	0.0158 km^3
Manual Approach	Subsidence	0.11888 km^3
	Uplift	0.0157 km^3

Table S2. Volumes estimation results: uplifted and subsided volumes obtained for the two method.

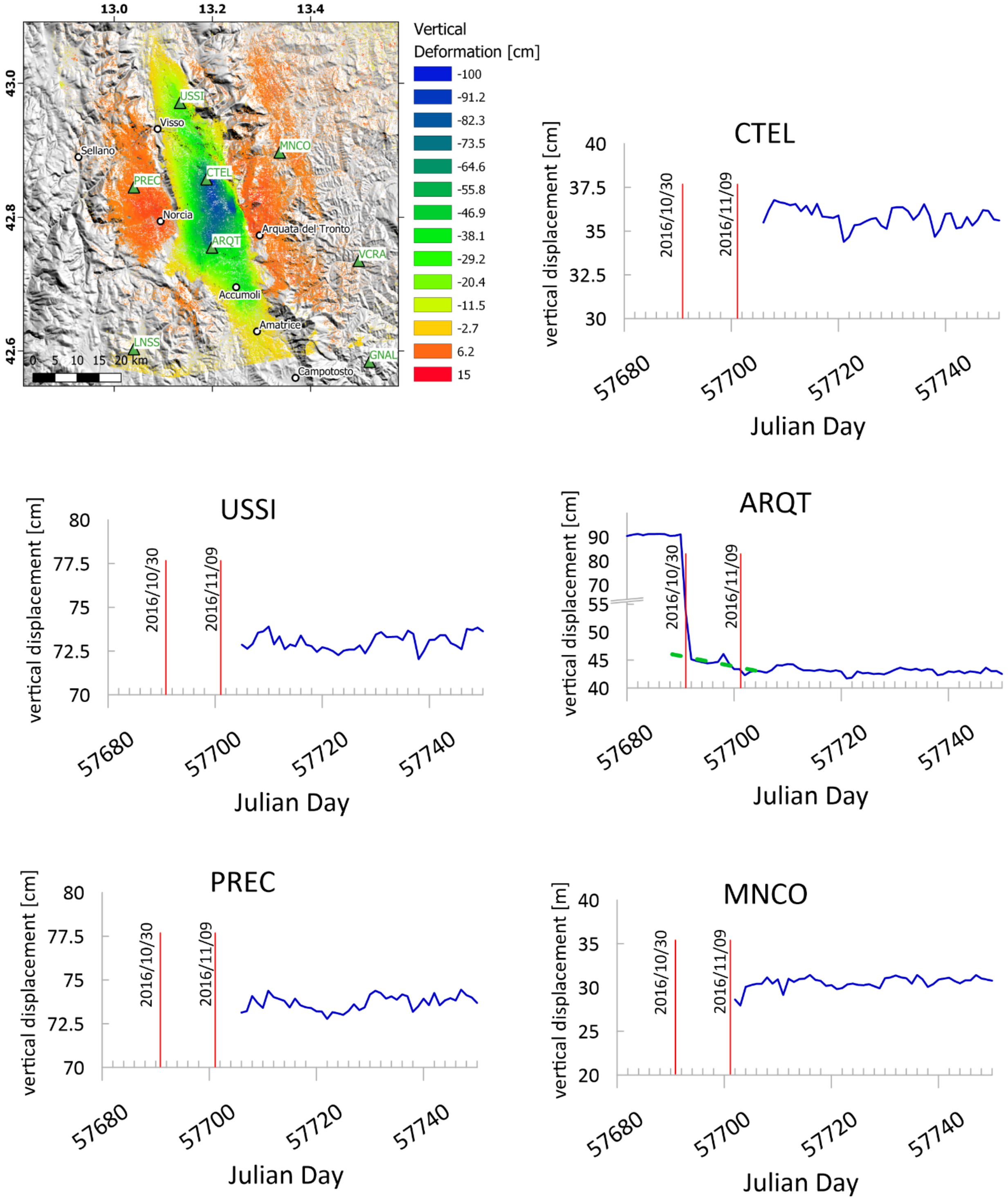

Figure S1. Continuous GPS data can help on understanding the kinematic of the deformation. The figure shows the map of displacement resulting from DInSAR technique, and some relevant GPS time series of measurement in the area imaged by SAR data. The plots show the vertical displacement of the GPS stations. The red vertical bars refer to the October 30, 2016 earthquake (left bar) and the date of last SAR image we exploited for the DInSAR analysis (November 9, 2016, right bar). We used GPS measures with the aims of retrieving any possible post-seismic deformation that can be included in the DInSAR map. Unfortunately, four of the five identified stations do not collected measurements soon after the main events and before the last SAR image. Only the ARQT receiver seems to show an early post-seismic signal, with a very gentle additional subsidence (see dashed green line in the corresponding plot) in the first 10 days after the Mw 6.5 Norcia earthquake.
